# Towards acoustic discrimination of tropical tuna associated with Fish Aggregating Devices

**DOI:** 10.1371/journal.pone.0216353

**Published:** 2019-06-05

**Authors:** Gala Moreno, Guillermo Boyra, Igor Sancristobal, David Itano, Victor Restrepo

**Affiliations:** 1 International Seafood Sustainability Foundation (ISSF), Washington DC, United States of America; 2 Sustainable fisheries management, Marine Research, Azti-Tecnalia, Pasaia, Gipuzkoa, Spain; 3 Sustainable Management of Fisheries, Collecte Localisation Satellites (CLS), Ramonville-Saint-Agne, France; 4 Fisheries consultant, Honolulu, Hawaii, United States of America; Instituto Portugues do Mar e da Atmosfera, PORTUGAL

## Abstract

Tropical tuna support some of the largest and most valuable artisanal and industrial fisheries worldwide, conducted to a large degree with Fish Aggregating Devices (FADs). Yellowfin, bigeye and skipjack are the main tuna species found in mixed aggregations around FADs and they are simultaneously encircled by the purse seining operation. One of the key challenges that purse seine fleets fishing with drifting FADs face in all oceans is to be able to target species in healthy condition such as skipjack, while reducing impacts on bigeye and yellowfin in areas where there is a need to reduce fishing pressure on these species. The present paper explores a technical solution for selective fishing at FADs by means of acoustic equipment used by purse seiners. Acoustic frequency response of skipjack and bigeye tuna were determined at 38, 120 and 200 kHz. Skipjack showed stronger response at higher frequencies. On the contrary, bigeye showed stronger responses at lower frequencies. The robust pattern shown in frequency responses of the two species demonstrates the potential to predict abundance and species proportions based on purely acoustic measures. The paper also addresses the conditions that need to be met to successfully apply this technology for selective fishing as well as other uses of direct acoustic observations to support tuna conservation.

## Introduction

Tropical tunas support some of the largest and most valuable artisanal and industrial fisheries worldwide [1]. Presently, on an industrial scale, tunas are mainly caught by purse seine, longline and pole-and-line gear over wide areas of the pelagic ecosystem. Of these fishing gears, the largest catch in terms of weight is taken by purse seine fisheries accounting for a significant portion of the income for many countries through employment, revenues from licensing and access fees, and economic spin-off [2]. Purse seine tropical tuna fisheries represent nearly USD 26 billion end value [3]. Around 65% of global tropical tuna purse seine landings result from fishing with Fish Aggregating Devices (FADs). Other forms of purse-seine fishing include setting on free swimming schools (FS) and in the Eastern Pacific Ocean (EPO) setting on schools associated with dolphins [4].

FADs are constructed by fishers and used to aggregate fish. FADs can be anchored or drift with currents. The industrial tropical tuna purse seine fleets around the world primarily fish on drifting FADs (DFAD). DFADs attract the 3 main tropical tuna species, skipjack tuna (*Katsowonus pelamis*), bigeye tuna (*Thunnus obesus*) and yellowfin tuna (*Thunnus albacares*) together with some non-target species that also aggregate around floating objects [5]. Skipjack landings represent more than half of the global catch of tunas and all major stocks are currently assessed in a healthy condition [6]. However, the yellowfin tuna stock in the Atlantic Ocean (AO) is overfished and, because recent catches have been excessive, it is possible that overfishing is also now occurring [7]. In the EPO, slight overfishing of yellowfin is taking place [8] and in the Western and Central Pacific Ocean (WCPO) the stock is in healthy condition. In the Indian Ocean (IO), yellowfin tuna is overfished, and overfishing has been occurring since 2015 [6]. While bigeye tuna stock is in healthy condition in the IO, the AO stock is overfished, and overfishing is occurring. Recent assessment of the WCPO bigeye stock showed a more optimistic status for bigeye, compared to prior assessments, indicating that overfishing is likely not occurring and that the stock is not being overfished. Finally, in the EPO, while the stock is not overfished, overfishing was occurring on average in recent years (2015–2017) [6].

Commonly, the three tropical tuna species can be found simultaneously in different proportions and sizes at a given DFAD depending on the region and time of the year [9,10]. In general, skipjack tuna represents 60–70% of DFAD-associated catch. The mean annual species composition of DFAD associated purse seine catches is similar across the four ocean basins: skipjack dominates the catch at DFADs (from 59% to 73% depending on the ocean) but was in many cases found with bigeye (15% to 25%) and yellowfin (7%-24%). However, regional differences do exist on the composition of species found at DFADs [9]. Skipjack are mostly caught at similar sizes, irrespective of whether they are from FS or DFAD associated schools. In contrast, the majority of yellowfin and bigeye caught on DFADs are small and mainly immature (<60 cm) in contrast to yellowfin and bigeye caught in FS, which are larger on average and mostly mature [9].

Increased efficiency for catching tunas due to use of DFADs, resulted in a shift in purse seine fishing strategy from FS or unassociated tuna fishing, to fishing on anchored FADs and DFADs. One of the key challenges that face purse seine fleets fishing on DFADs in all oceans is to be able to target species for which stocks are known to be in healthy condition such as skipjack while reducing their impact on bigeye and yellowfin stocks in regions where there is a need to conserve of these species. Given the fact that the entire DFAD aggregation is encircled and captured during the purse seine operation and the three species may be present, only a change in fishing technology could address this problem [11].

### The use of acoustics in FAD fishing

**Fig 1:**
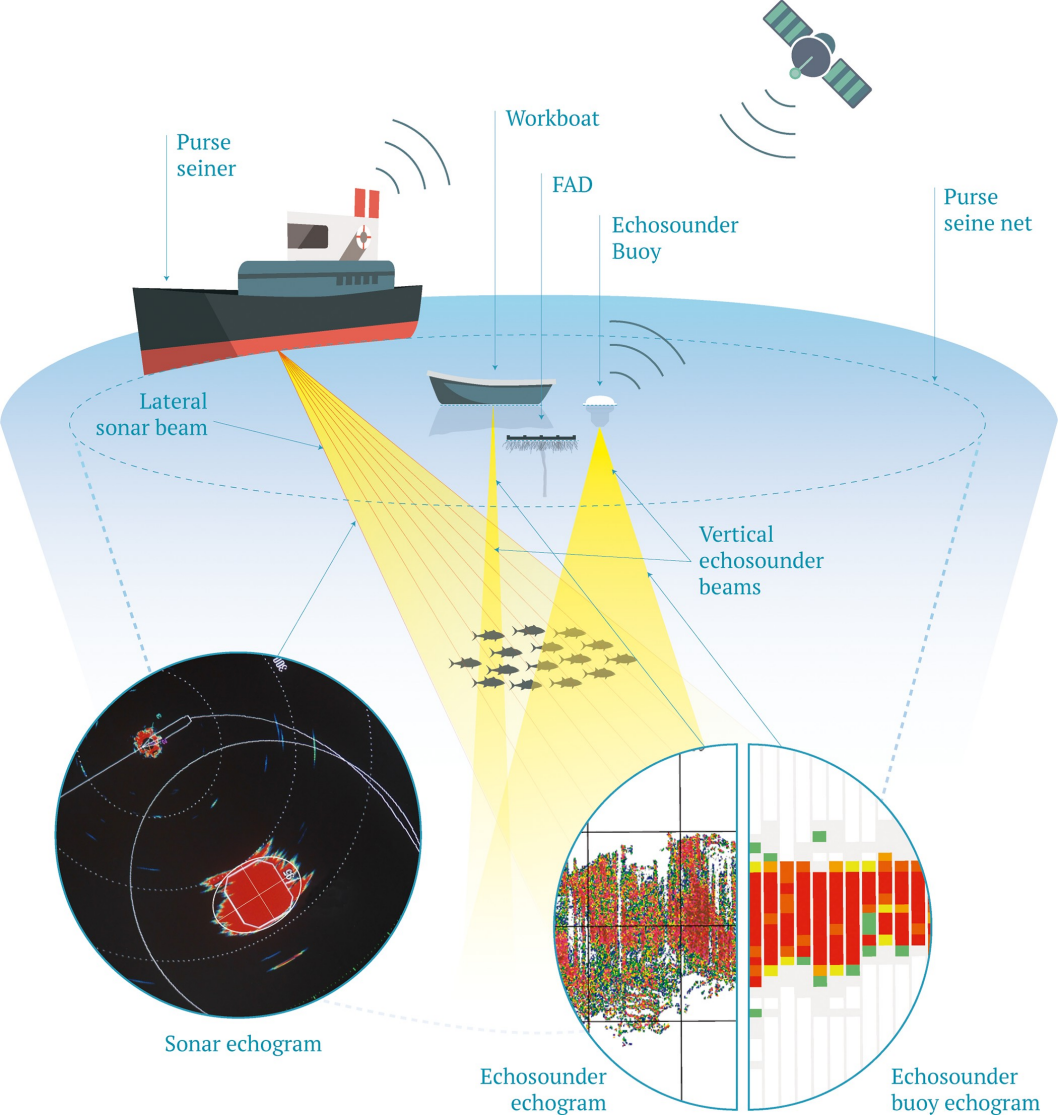
Synoptic diagram describing the collection of different type of acoustic data from tuna purse seiners (lateral sonar beam, echo-sounder from the work-boat and echo-sounder of the buoy used to track DFADs) as well as the echogram associated to each tool.

Acoustic technology represents an indispensable fishing tool that purse seine vessels targeting tropical tunas use to detect tunas, evaluate school size and position and assist in making the set (Fig 1). Purse seiners began to introduce electronic devices into their fishing operations (e.g. bird radar, navigation radar, underwater current meters, sonar, etc.) to improve catches on free schools [12]. Key technological innovations that have been adopted by purse seine fleet through the early 2000’s were documented in the European Union (EU) research project ESTHER [13]. Since then, further technological innovations have continued to increase efficiency of the fleet, especially for the development of DFAD fishing as documented in the EU research project CECOFAD [14].

Purse seiners are equipped with long-range sonar that can effectively scan out to around two nautical miles to investigate free swimming and DFAD associated tuna schools. If an aggregation is present the range can be reduced with increased definition or a short-range sonar used to assure the purse seine encircles the whole school or the majority of the aggregation. If fishers are not able to confirm enough tuna with sonar to conduct a set, they may approach the DFAD with the echo-sounders onboard or on smaller auxiliary craft to check the aggregation. These echo-sounders can allow both vertical and oblique scanning and operate at different frequencies that may reveal additional information to the fishers. The investment in acoustic equipment shows the importance of this technology for fishers. The most advanced purse seine vessels, in terms of technology, use two long-range sonars costing over 200,000 € each (Simrad, SU90), two short-range sonars costing over 200,000 € each (Simrad, CS94), and up to four echo-sounders (one Simrad ES80 with vertical beaming and three with oblique beaming) costing around 40,000 € each. The most advanced vessels have incorporated ultra-large range sonars (Simrad, ST90) costing nearly 300,000 € and multibeam sonars (Simrad, CS90) of around 115,000 € (Lleches, M.A., Simrad, 2019, pers. comm.). These examples correspond to one of the main brands used by fishers, the other brand, which is also widely used is Furuno but costs do not differ significantly. Overall, the acoustic equipment onboard alone could exceed one million euros.

In the past 15 years, introduction and refinement of advanced acoustic technology allowing remote detection of tuna schools on DFADs has radically changed the fishing strategy of trips and the way fishers decide which fishing grounds and DFADs they visit. Currently almost 100% of the buoys used to track DFADs in large purse seiners are equipped with an echo-sounder that provides remotely via satellite, an estimation of the biomass aggregated underneath the DFAD [15,16]. Each purse seine vessel can simultaneously monitor up to ~400 DFADs scattered in different fishing zones. Those DFADs can also be monitored from shipowner offices and supply vessels. The challenge is to make a route linking the largest number of productive DFADs as possible, minimizing long routes to a single DFAD, as fuel consumption is a major contributor to the overall operating costs of fishing vessels [17,18]. Fishers plan visits to DFADs based mainly on the remote biomass information provided by these echo-sounder buoys, their historic catch records in the different areas and seasons, and the information provided by other fishers [19].

Currently, there are 3 different echo-sounder buoy brands widely used in the tuna purse seine fishery worldwide, Zunibal, Satlink and Marine Instruments. These buoys operate with lower frequencies of 38 kHz in some buoys and up to 200 kHz in others. The range of maximum depth at which biomass is recorded varies from 115 to 150 m (Table 1). None of the echo-sounders, including dedicated sounders installed onboard purse seiners nor those integrated with GPS satellite buoys used to track DFADs, have the capability to directly identify fish size and species. Although buoy companies are working towards acoustic discrimination of tuna and have improved the hardware for that, discrimination is not possible yet. Consequently, vessels can navigate to a group of DFADs over long distances, only to discover that the aggregation is formed by small-sized or undesired species of tuna. In this situation, it is difficult for captains to avoid setting the net, even if the resulting catch is expected to be less than ideal, as some income must be gained to offset fuel costs and expenses. Fuel consumption can vary from 10.000–20.000 l/day depending on the vessel size and distance covered. If echo-sounder buoys had the ability to discriminate the species and sizes of tunas found at DFADs, fishers could avoid navigating to areas where non-desired species and sizes of tunas represent the majority of the catch. Likewise, the use of onboard sonar and echo-sounders capable of tuna discrimination would allow more accurate evaluation of the species and sizes present at DFADs, allowing fishers making more sustainable decisions.

**Table 1:** Characteristics of the most used echo-sounder buoy models used to track DFADs.

Buoy Brand	Zunibal	Zunibal	Satlink	Satlink	Marine instruments	Marine instruments
Buoy Model	Tuna 8 Explorer	Tuna 8 Xtreme	ELB3010 ISL	ISD+	M3I	M3I+
**Frequency**	120 kHz	120 kHz	190,5 kHz	38 kHz /200 kHz	50 kHz	50 kHz/200kHz
**Beam angle**	22º	22º (low Q transducer)	32º	32º	35º	42º (50kHz) /8º (200kHz)
**Power (W)**	200 watts	200 watts	120 watts	200 watts	500 watts	500 watts
**Blind area (m)**	3 m	3 m	3 m	3 m	6 m	3 m
**Max depth (m)**	120 m	120,6 m	115 m	115 m	150 m	150 m
**Depth layers**	75 (1,6 m resolution)	67 (1,8 m resolution)	10 (11,5 m resolution)	10 (11,5 m resolution)	50 (3 m resolution)	50 (3 m resolution)
**Ping rate (echo-sounder sampling frequency)**	1 min	20 seg	15 min	15 min	5 min	1 min
Biomass index*	Biomass in tons (dB for each layer	Biomass in tons (dB for each layer)	Biomass in tons derived from SKJ density	% Biomass in tons derived from SKJ BET YFT density	Integers from 0 to 7 for each layer	Integers from 0 to 7 for each layer

*Information provided by the buoy company. Not clear how biomass is calculated

The acoustic data needed to discriminate tuna species using acoustic gear is not yet available, as few studies have addressed acoustic properties of tropical tunas. This may be due to the fact that tropical tunas are usually found in offshore fishing grounds and DFAD aggregations are ephemeral, making the research expensive and logistically difficult. Concerns regarding impact of DFADs on tuna stocks and on the ecosystem are relatively recent [20, 21] and acoustic tools have not yet been considered as a tool to support sustainability of the fishery.

There is great value in understanding acoustic properties of tropical tuna, as species-specific target strength (TS) measurements are needed to allow the scaling of signal strength to biomass and to accurately identify species and discriminate size. Despite the importance of characterizing TS of tropical tuna species, no consistent TS-length and TS-frequency relationships have been determined, as measurements to date have generally been made with only a single frequency [22–25]. Only recently has there been focus on this avenue of research as leading toward a solution to selectively decrease mortality of certain species and sizes of tunas at DFADs [26]. Acoustic properties have long been used when assessing other (non-tuna) species for direct estimates of abundance [27] as well as species discrimination [28], for improved selectivity. A need to reduce fishing mortality on certain tropical tuna stocks and to develop more selective fisheries, leads us to place emphasis on testing utility of acoustic discrimination of tropical tuna. Our research has the aims of developing a technological tool to achieve such reductions as well as to gather species-specific abundance data to support stock assessment and DFAD behavioral studies [29–31].

One way to discriminate tropical tuna species using acoustic equipment found onboard tuna purse seine vessels takes advantage of the fact that, bigeye and yellowfin tuna have a swimbladder, whereas skipjack do not. The highest contribution to the acoustic response comes from the gas inside the swimbladder (when present) [23]. There is a strong contrasting response to different acoustic frequencies, between species with and without swimbladders [32, 33]. This differencing could be used to acoustically distinguish skipjack from bigeye and yellowfin tunas and therefore improve targeting to desired species, if discrimination algorithms making use of different frequencies are developed.

This paper addresses acoustic discrimination of the main tropical tuna species at DFADs, with the following specific objectives:

- At an operational level, establishing the basis for collection of acoustic data from a purse seine fishing vessel in a regular trip, without disturbing the fishing operation. These would allow further acoustic studies in a cost-effective way.
- Determine acoustic frequency responses of skipjack and bigeye tuna and providing outcome of a first attempt to discriminate skipjack from yellowfin and bigeye (combined), based on information collected with echo-sounders working at 3 different frequencies.
- Discuss conditions that need be met to successfully apply this technology for conservation of tropical tunas.

## Materials and methods

### Data collection

The data analyzed here were obtained during 2 scientific cruises organized by the International Seafood Sustainability Foundation (ISSF). The first in May 2014 was conducted in the Central Pacific Ocean onboard *ALBATUN TRES* a 115 m and 4,406 GT Spanish-flagged purse seiner. The cruise departed Christmas Island (Kiribati) on May 3rd and returned to Tarawa (Kiribati) on May 31^st^. The second cruise was conducted in equatorial Atlantic waters during March and April 2016, onboard the purse seiner *MAR DE SERGIO*, an 83m, 2.767 GT (~1.300 t carry capacity), Spanish-flagged vessel. This cruise departed Abidjan (Ivory Coast) on March 14^th^ and returned to Dakar (Senegal) on April 11th.

For both cruises, the acoustic equipment was mounted in an 8-meter work-boat called a “panguita” which was deployed from the purse seiner before the purse seine net was set at DFADs. Transducers were focused vertically downwards, in order to acoustically survey fish aggregations to 200 m below the surface. Acoustic data were collected with a SIMRAD EK60 echo-sounder connected to 38 kHz, 120 kHz and 200 kHz split-beam transducers, used with a pulse duration of 0.512 ms (Table 2). Frequency calibration was conducted at the beginning of each cruise, following the standard procedure with a tungsten carbide sphere of 38.1 mm [34]. Acoustic and navigation data were stored electronically on a PC through SIMRAD ER60 software.

**Table 2:** Configuration of the acoustic equipment and calibration parameters.

**2014 Pacific Ocean**			
**Frequency (kHz)**	38	120	200
**Pulse duration (us)**	512	512	512
**Power (W)**	2000	250	150
**Gain (dB)**	26.16	25.96	27.09
**Sa Correction (dB)**	-0.86	-0.39	-0.34
**Ath. Beam Angle (deg)**	6.92	6.38	6.43
**Along Beam Angle (deg)**	6.94	6.39	6.37
**Ref. target Target Strenght (TS) (dB)**	-42.3	-40	-39.9
**TS deviation (dB)**	5	5	5
**Root Mean Square (RMS) beam model**	0.19	0.18	0.2
**RMS polynomial model**	0.16	0.16	0.15
**2016 Atlantic Ocean**			
**Frequency (kHz)**	38	120	200
**Pulse duration (us)**	512	512	512
**Power (W)**	2000	250	150
**Gain (dB)**	25.83	26.46	26.88
**Sa Correction (dB)**	-0.8	-0.38	-0.3
**Ath. Beam Angle (deg)**	6.79	6.38	6.43
**Along Beam Angle (deg)**	6.47	6.35	6.37
**Ref. target TS (dB)**	-42.3	-40	-39.9
**TS deviation (dB)**	5	5	5
**RMS beam model**	0.46	0.28	0.35
**RMS polynomial model**	0.39	0.25	0.33

During each cruise, in order to conduct acoustic surveys without disturbing the fishing operation, the panguita was attached to the DFAD about 10 minutes prior to the beginning of the sets and remained attached during the entire setting operation. During the first part of the set, the panguita drifted with the DFAD and, afterwards, it moved slowly to keep the DFAD separated from both the net boundaries and the purse seiner as the net was pursed.

Each time acoustic EK60 data was recorded sampling of the catch was conducted. Approximately 0.8 tons of catch during the first cruise and between 1 and 2 tons per set during the second cruise were sampled per sets for which acoustic surveys were conducted. Tuna individuals were “spilled” into a fiberglass box of 110 cm x 70 cm x 100 cm dimensions to obtain an unbiased, random sample during the fish loading process and repeated depending on the total amount of fish captured during the set. The samples were recorded to species and caudal fork length. We assumed that the catch for a particular purse seine set corresponded to the actual biomass of tunas that were present at that DFAD.

### Data analysis

Simrad EK60 echo-sounder data were pre-processed using Echoview (Myriax inc.) software. The acoustic data were processed until the purse seine net started to be visible at the lower part of the echogram. The pre-processing excluded data from the upper (25 m) and lower (150 m) depths as were signals with acoustic spikes (interferences). Then, in order to isolate fish echoes, we followed the procedure used by Boyra et al. (2018), applying a school detection algorithm [35] to retain the main aggregation (attributed to tuna) while the echoes outside the aggregation (considered plankton and/or micronekton) were rejected. After smoothing by a 5x5 convolution, “acoustic schools” (i.e., the main aggregation around the DFAD) were selected using: minimum total school length and height of 0.2 m; minimum candidate length and height of 0.1 m; and maximum vertical and horizontal linking distances of 5 and 20 m, respectively.

#### Determination of frequency response of tropical tuna species

In order to obtain the frequency response patterns for each tropical tuna species, we selected monospecific or nearly monospecific catches at DFADs, *i*.*e*., those having a percentage in weight of one of the tuna species of at least 90% (Table 3). The mean frequency response was obtained computing Mean Volume Backscattering Strength (MVBS) [30], echo-integrated in cells of 100 pings and 25 m depth bins, and then obtaining differences of MVBS between each high frequency “i” and 38 kHz (ΔMVBSi-38).

**Table 3:** Summary of the catches of the main tuna species at each DFAD, indicated by set code (year and set number). Percentages per individuals of each species as well as percentages per weight (indicated with a “w”), mean (L) and standard deviation of the length per species (sdL), total catch and total number of sampled individuals (N) per set are presented. (SKJ: skipjack; BET: bigeye; YFT: yellowfin).

set	date	SKJ	BET	YFT	SKJ.w	BET.w	YFT.w	L.SKJ	L.BET	L.YFT	skj.sdL	bet.sdL	yft.sdL	L.mean	Catch	N
(yynn)	(dd/mm/yyyy)	(%)	(%)	(%)	(%)	(%)	(%)	(cm)	(cm)	(cm)	(cm)	(cm)	(cm)	(cm)	(tons)	
1404	5/6/2014	75	18	8	55	34	11	41	54	51	0.24	0.52	0.60	43.7	40	914
1405	5/7/2014	35	56	9	11	85	4	46	73	51	0.28	1.14	0.90	61.7	78	384
1406	5/8/2014	5	94	1	1	99	0	47	75	54	1.63	1.08	3.89	73.2	25	186
1407	5/9/2014	21	75	5	7	92	2	46	68	51	0.69	0.67	1.93	62.5	95	499
1408	5/10/2014	53	36	12	25	66	8	44	65	51	0.22	0.73	0.48	52.3	140	1077
1409	5/11/2014	54	37	9	26	68	6	45	66	51	0.25	1.04	0.63	53.2	40	449
1411	5/12/2014	47	40	13	30	60	10	46	59	50	0.41	0.76	0.66	51.8	20	290
1412	5/13/2014	87	7	6	77	12	11	46	56	54	0.23	1.06	2.96	46.9	20	424
1413	5/14/2014	82	13	5	66	27	8	44	56	52	0.27	0.48	1.32	45.7	55	932
1414	5/15/2014	48	47	5	28	68	5	48	63	55	0.32	0.67	2.82	55.3	75	606
1415	5/16/2014	69	27	4	49	46	4	48	62	55	0.45	1.01	2.54	51.8	55	523
1416	5/17/2014	55	40	5	21	73	6	46	73	56	0.49	1.43	7.01	57.1	60	289
1417	5/18/2014	85	8	7	38	56	6	45	56	52	0.12	0.75	0.61	46.5	180	1038
1418	5/19/2014	54	40	7	25	71	4	45	66	51	0.40	1.28	0.88	53.6	65	375
1420	5/20/2014	48	45	7	27	68	5	46	60	52	0.18	0.48	0.52	53	215	1082
1422	5/22/2014	57	20	23	44	29	26	43	51	49	0.27	0.56	0.35	45.8	110	767
1424	5/24/2014	99	1	0	100	0	0	48	32	49	0.32	0.72	1.41	48.3	170	636
1426	5/26/2014	98	2	1	94	4	2	52	58	62	0.34	9.63	18.93	52.1	125	358
1427	5/27/2014	96	2	2	94	4	2	49	55	47	0.29	4.77	1.86	49	170	617
1624	4/1/2016	38	34	28	17	65	18	47	69	53	0.70	1.83	1.05	56.3	10	158
1625	4/1/2016	59	23	18	42	30	27	49	59	60	0.73	0.90	3.68	53.3	10	123
1626	4/1/2016	67	12	21	58	17	25	46	53	51	0.25	1.08	0.62	47.9	15	313
1627	4/2/2016	90	1	10	86	1	12	49	58	53	0.19	0.47	1.31	49.3	45	383
1628	4/2/2016	90	2	9	87	2	11	48	51	51	0.12	3.62	0.47	48.2	55	456
1631	4/5/2016	62	2	36	43	1	36	47	46	52	0.37	2.71	0.75	48.7	20	291
1633	4/7/2016	65	30	5	32	64	4	45	53	43	0.68	0.61	1.25	47.1	10	315

#### Determining tuna biomass by species from acoustic parameters

In order to determine the utility of purely acoustic data to estimate biomass of tropical tuna by species at DFADs, the relationship between various acoustical parameters and the abundance, species distribution and mean size of tuna captured in all sampled DFADs was used. For those analyses both, mono-specific and multi-species aggregations at DFADs were studied. For each set, the modelled response variables were: (i) the total tuna catch, as well as the catches per tuna species as estimated by the skipper and the crew; (ii) the proportion of each tuna species, estimated through the spill sampling, both by weight and by number of individuals, and weighted by the abundance of each DFAD; and (iii) the overall mean tuna length and the mean length by tuna species, also obtained from the spill sampling. The acoustic parameters used as explanatory variables were: (i) the mean volumetric acoustic density (MVBS_i_) at each frequency i = 38, 120 and 200 kHz, echo-integrated in cells of 100 pings and 25 m depth bins, plus MVBS_MF_, the average of this parameter across the three frequencies; (ii) the mean and coefficient of variation of Nautical Area Scattering Coefficient at each frequency (NASC_i_), echo-integrated in the same cells and hence equivalent to the MVBS values integrated over the depth [36]; (iii) the frequency response (ΔMVBS_i-38_) as defined above; (iv) the mean depth of the aggregation for all the pings in the echogram, weighted to the NASC of each cell, as a proxy of the vertical distribution of tuna species at each DFAD. For yellowfin tuna, given that swimbladder development has been reported to occur only after reaching a certain minimum body size [37], the mean length of yellowfin tuna was also used as independent variable to try to explain the relationship between proportion of this species and frequency response.

Single and multiple linear (LM) and generalized linear models (GLM) were used to explore the relationships between the variables. GLMs were applied with proportional dependent variables, in order to account for non-linearity and heteroscedasticity of this type of data, using a binomial family, plus “probit” and “logit” link functions for the independent variables [38, 39]. For the rest of the dependent variables, regular linear models with gaussian errors were used. Model selection was done by AIC (Akaike Information Criterion) [40]. As absolute measure of goodness of fit, ordinary R^2^ was calculated for LMs and McFadden R^2^ [41], for GLMs. Statistical analyses were carried out with R [42].

## Results

The protocol established for acoustically surveying aggregations at DFADs from a purse seine vessel during a regular fishing trip permitted both having access to DFADs belonging to the fishing company and gathering scientific acoustic data. Acoustic samples were taken just before and during the fishing set which allowed in each set around 60 minutes of acoustic data collection without disturbing the fishing operation.

### Frequency response of tropical tuna species

During the first cruise, among 27 sets carried out on the trip, three sets on DFADs (numbers 1424, 1426 and 1427) had a percentage of skipjack above 95% (Table 3) and were selected for the frequency response analysis of this species. The intensive spill sampling provided mean sizes of skipjack of 48.4, 51.9 and 48.9 cm respectively for the three sets. In addition, aggregations with more than 90% bigeye tuna where found at 2 DFADs (set numbers 1406 and 1407, Table 3) and those sets were selected for analysis on frequency response of bigeye tunas. Mean sizes of bigeye tuna at these DFADs were 75 and 68 cm respectively.

**Fig 2:**
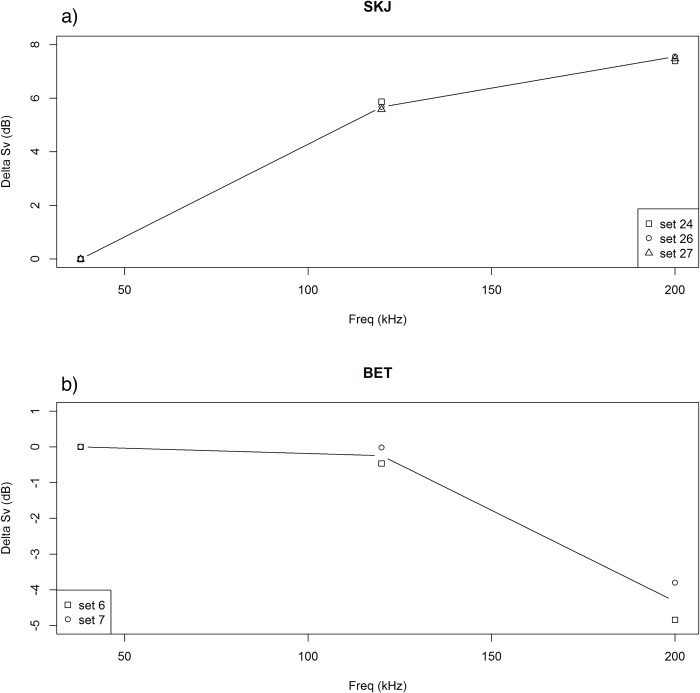
Frequency response of a) skipjack tuna and b) bigeye tuna at 38, 120 and 200 kHz frequencies.

**Fig 3:**
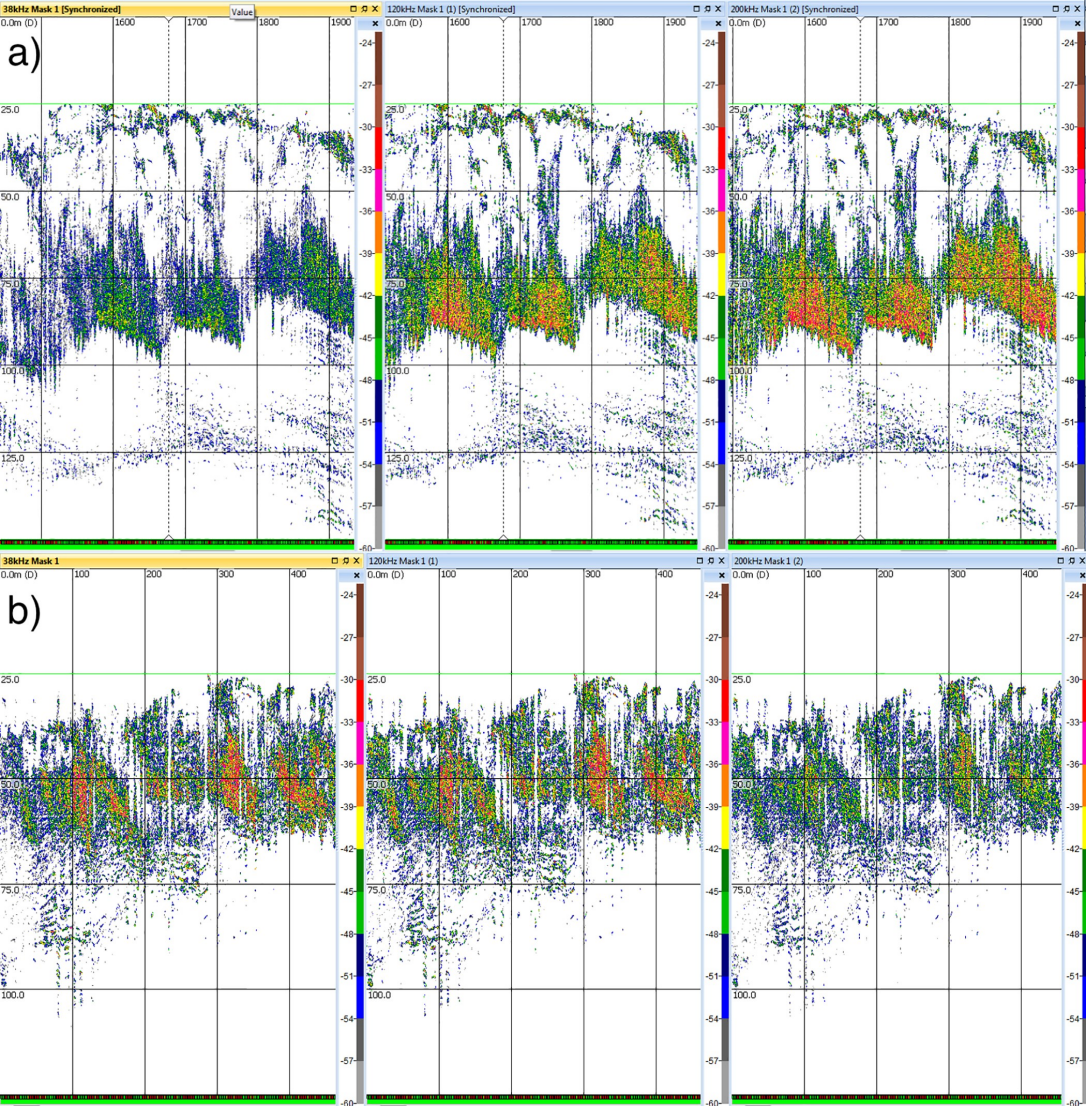
Example echograms showing the frequency response of a) skipjack and b) bigeye at 38, 120 and 200 kHz frequencies.

Skipjack tuna showed frequency response patterns typical of bladderless species in general (Figs 2 and 3). The differences were over 7 decibels higher at 200 kHz than at 38 kHz, and almost 6 decibels higher at 120 kHz compared to 38 kHz. This was a consistent pattern for the 3 pure skipjack aggregations observed (Fig 2A). In contrast, bigeye tuna showed a stronger response at lower frequencies. The differences were less than 1 dB between 38 kHz and 120 kHz and over 3 dB between 38 kHz and 200 kHz (Fig 2B).

### Determining tuna biomass by species from acoustic parameters

**Fig 4:**
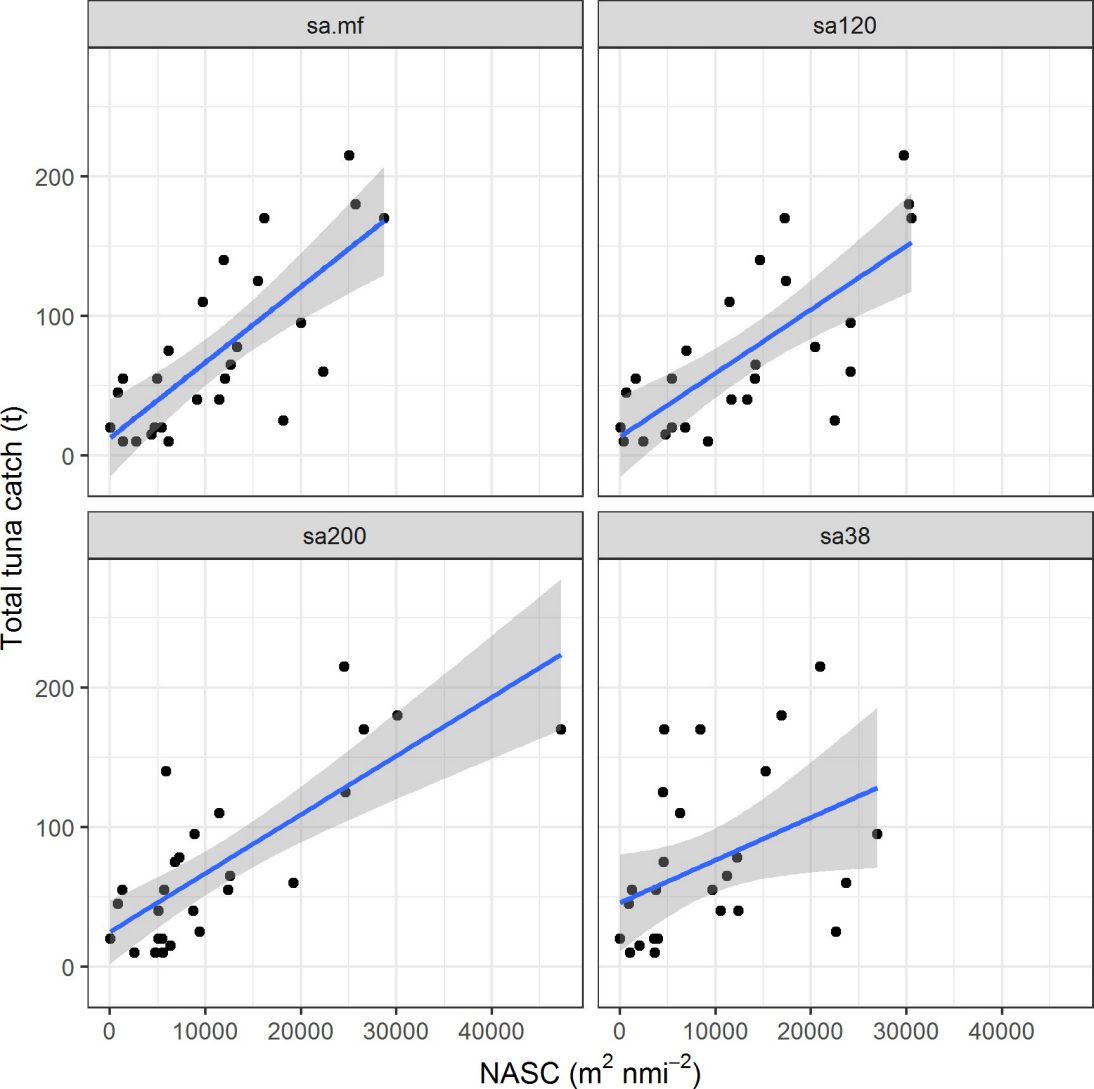
Scatterplot of total catch against (a) NASC_MF_, (b) NASC_120_, (c) NASC_200_ and (d) NASC_38_.

Concerning biomass abundance prediction, total tuna catch was significantly correlated with both NASC and MVBS at all frequencies (Table 3 and Fig 4). In general, NASC performed better than MVBS, probably due to the integrative nature of the surface acoustic measure, which includes implicitly information on the vertical range of the aggregation, while the MVBS reports only acoustic density.

In general, total catch correlated better with acoustic backscattering measures at 200 kHz than 120 kHz, probably because of the higher overall proportion of skipjack across DFADs (around 70%) and the higher response of this species to higher frequencies (especially the 200 kHz, Fig 2). Addition of mean depth of the aggregation to NASC significantly improved the prediction of total catch (Table 4).

**Table 4:** Characteristics and results of the most relevant models tested. (Significance codes for the slope coefficients: 0 ‘***’ 0.001 ‘**’ 0.01 ‘*’ 0.05 ‘.’ 0.1 ‘ ‘ 1).

Dependent	Independent	Type	Family	Link	Weight	Intercept	Slope(s)			R^2^	AIC
Catch	NASC_38_	LM	Gaussian	Identity	-	45.8	0.0031 *			55	287
	NASC_120_					13.5	0.00456 ***			54	271.6
	**NASC**_**200**_					**24.8**	0.00421 ***			**60**	**267.7**
	NASC_MF_					12.7	0.0054 ***			57	270
Catch	MVBS_38_	LM	Gaussian	Identity	-	109	0.35 *			20	285.7
	MVBS_120_					108.5	0.35 *			21	285.6
	MVBS_200_					109	0.36 *			21	285.5
	MVBS_MF_					108.7	0.36 *			21	285.6
Catch	NASC_38_ + z_mean_	LM	Gaussian	Identity	-	-100	0.0019.	2.6 ***		65	266.5
	NASC_120_ + z_mean_					-82	0.0029 ***	1.9 ***		75	257.8
	NASC_200_ + z_mean_					-61	0.0027 **	1.67 **		73	259.1
	**NASC**_**MF**_ **+ z**_**mean**_					**-78**	0.0035 ***	1.8 ***		**75**	**257.4**
Catch_skj_	NASC_38_	LM	Gaussian	Identity	-	0.41	4.7E-06			1	38.2
	NASC_120_					0.1	0.00002 *			18	33.3
	**NASC**_**200**_					**0.0015**	0.000033 ***			**64**	**12.1**
	NASC_MF_					0.0065	0.000027 *			24	31.3
Catch_BET_	**NASC**_**38**_	LM	Gaussian	Identity	-	**-5.50E-04**	0.000035 ***			**53**	**9.5**
	NASC_120_					-0.016	0.000025 ***			40	15.5
	NASC_200_					0.21	8.7E-06			6	27.2
	NASC_MF_					0.014	0.000027 **			34	18
Catch_YFT_	NASC_38_	LM	Gaussian	Identity	-	0.045	0.0000004			0	-70
	NASC_120_					0.042	0.0000005			1	-70.1
	NASC_200_					0.045	0.0000004			0	-70.1
	NASC_MF_					0.043	0.0000006			1	-70.1
Catch	ΔMVBS_200-38_	LM	Gaussian	Identity	-	69.7	2.50			2	291
	ΔMVBS_120-38_					56	9.10			9	288.9
%_SKJ_	ΔMVBS_200-38_	GLM	Binomial	Logit	Catch	0.44	0.27 ***			51	347.6
	ΔMVBS_200-38_			**Probit**		**0.26**	0.16 ***			**52**	**339.2**
%_SKJ_	**ΔMVBS**_**200-38**_	GLM	Binomial	Probit	Catch	**0.26**	0.16 ***			**52**	**339.2**
	ΔMVBS_120-38_					-0.12	0.29 ***			44	395.2
%_SKJ.w_	ΔMVBS_200-38_	GLM	Binomial	Probit	Catch	-0.34	0.2 ***			56	447.4
**%**_**SKJ**_						**0.26**	0.16 ***			**52**	**339.2**
%_BET_	**ΔMVBS**_**200-38**_	GLM	Binomial	Probit	Catch	**-0.51**	-0.17 ***			**50**	**346.4**
	ΔMVBS_120-38_					-0.09	-0.3 ***			44	402
%_YFT_	ΔMVBS_200-38_	GLM	Binomial	Probit	Catch	-1.41	-0.04 ***			7	172.9
	ΔMVBS_120-38_					-1.28	-0.08 ***			7	172.4
%_YFT_	ΔMVBS_200-38_	GLM	Binomial	Probit	Catch	-1.41	-0.04 ***			7	172.9
	ΔMVBS_200-38_ + L_yft_					0.1	-0.04 ***	-0.029.		8	171.9
	**ΔMVBS**_**200-38**_ * L_**yft**_					**3.33**	-0.63 *	-0.09 **	0.01 *	**11**	**168.6**
%_SKJ_	ΔMVBS_200-38_	GLM	Binomial	Probit	Catch	0.26	0.16 ***			52	339.2
	ΔMVBS_200-38_ + %_YFT_					**0.45**	0.16 ***	-2.15 ***		**55**	**322**
L_tot_	ΔMVBS_200-38_	LM	Gaussian	Identity	-	53.25	-0.83 *			21	168.8

Regarding biomass abundance of individual species, catches of bigeye and skipjack correlated significantly with both NASC and MVBS at most frequencies. As for total tuna biomass, best results were obtained with NASC (Table 4). The prediction models showed significant correlations (p < 0.001) and moderate coefficients of determination (up to 60%). Prediction of yellowfin catches were non-significant (perhaps due to low proportions of this species through the sets). Catches of skipjack correlated better with the highest frequencies (200 and 120 kHz) and catches of bigeye with the lowest frequencies (38 and 120 kHz), in clear agreement with the frequency response of each species (Fig 2). In general, high frequencies (120 and 200 kHz) better predicted skipjack abundance and the lower frequency (38 kHz) better predicted bigeye tuna, whereas the synthetic multifrequency averages NASC_MF_ and MVBS_MF_ were less selective than the individual frequencies, obtaining reasonably good (highly significant) correlations for any species at the cost of scoring lower than the best individual correlations.

**Fig 5:**
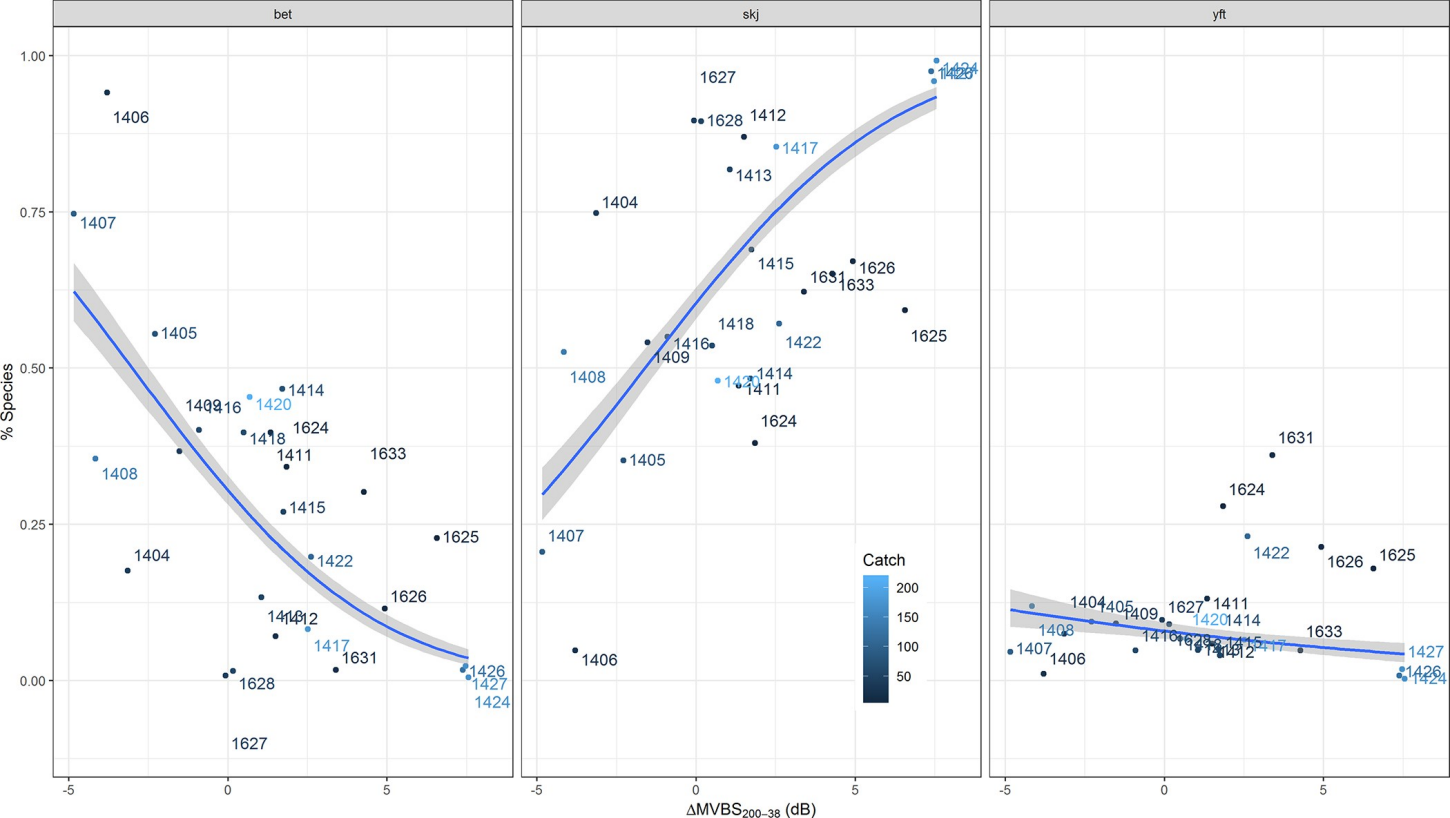
Scatterplots of percentage of the three main tuna species against frequency response ΔMVBS_38-200_.

Concerning the prediction of species proportions, frequency response was significant in predicting (p < 0.001, Table 4) proportions of the three species, although as shown in the scatterplots (Fig 5) most of the points were outside the confidence intervals. The scores were better when predicting proportions by individuals instead of by weight. In general, both transformations used in the GLMs (probit and logit) provided similar scores, although probit provided consistently better agreements. Note that, in the table and Figs, most parameters with low scores are omitted for synthesis.

From the two available frequency response measurements, ΔMVBS_38-200_ performed better than ΔMVBS_38-120_ for all species (Table 4). For skipjack (as expected, based on its frequency response patterns) the predicted proportions increased for higher ΔMVBS, whereas for bigeye the pattern was the opposite, both species showing rather steep slopes in opposite directions (Fig 5). Interestingly, there was a significant correlation also for yellowfin tuna, which gave response similar to the pattern of bigeye, but with a flatter slope. When adding the mean yellowfin length to the model of yellowfin proportion, the model improved, largely due to the interaction between length and frequency response. Finally, no significant and relevant models were identified for prediction of mean size by species. Only mean tuna length could be significantly predicted (p < 0.05) using the frequency response data.

## Discussion

### Suitability of tuna purse seiners for gathering acoustic data

The scientific equipment onboard the workboat provided calibrated acoustic data to study frequency response of tropical tunas. In addition, the ability to encircle and capture nearly the entire tuna aggregation during each set made verification of acoustic targets highly reliable, thus reducing one of the main sources of uncertainty when assessing the presence of different species and sizes of tunas at DFADs. These circumstances provided data of a quality not possible to achieve when sampling from gear types that take only a small proportion of the entire school such as pelagic trawl, the most common gear sampled with acoustic equipment. For this work, we have assumed that the catch for a particular purse seine set corresponded to the actual biomass of tunas that were present at that DFAD. We consider this a reasonable assumption, because of the large size of the purse seine net and the fact that tuna tend to aggregate closer to the DFAD during a fishing operation. An industrial purse seiner’s standard net dimension is 1600–1800 m length and 250–320 m vertical drop which forms a set diameter of 550–580 m. Previous acoustic studies at DFADs have shown that more than 90% of the aggregated schools were within a 400 m radius from the DFAD [43]. In addition, in cases when a considerable amount of tuna evaded the set, they were usually well noted by the powerful array of acoustic equipment onboard and can be accurately accounted for in the calculations and/or to justify exclusion of that set from the analysis. We did not observe tuna evading or escaping sets during the cruise either using acoustics or visually. In summary, one of the results of this study is that tropical tuna purse seiners operating in a commercial mode constitute suitable platforms to study the acoustic properties of tunas. *In situ* acoustic research on tropical tunas has been rare until now. The methodology presented here opens a line of opportunities to achieve faster growth of knowledge on the acoustic properties of tropical tunas and the use of commercial operations to support science.

### The use of acoustics to estimate tropical tuna biomass by species

#### Frequency response patterns of tropical tuna species

In this work we were able to study frequency response patterns of two important tropical tuna species: bigeye and skipjack tuna. Our results present drastically different frequency responses between skipjack (without swimbladder) and bigeye (with swimbladder) (Fig 2). The results for skipjack are in agreement with the general trends observed for Atlantic mackerel [33,44–46], another well-known, bladderless scombrid species. Nevertheless, we found that ΔMVBS_38-120_ of skipjack was larger than that of mackerel. The cause of this difference is unknown but could be attributed to the size difference between the two species [26,47].

Concerning frequency response of bigeye tuna, the patterns obtained are consistent with the ones observed for other fishes with swimbladders [44] and observed in other large physoclists [48]: higher response at lower frequency. In this case, the decline of response was steeper from 120 to 200 kHz than from 38 to 120 kHz (Fig 4). The contrasting frequency response patterns for these species opens the potential for acoustic discrimination between them. During our cruises, mono-specific schools of yellowfin tuna at DFADs were not found, thus it was not possible to obtain the isolated frequency response of this tuna species. We have initiated efforts for obtaining yellowfin tuna frequency response and TS, as we explain below.

#### Potential of acoustic data to estimate overall tuna biomass and species´ proportion at DFADs

Concerning abundance, the estimation of the overall biomass for all the species found at a given DFAD together, the significant relationships between acoustic backscattering and the overall catches as well as catches of the two most abundant species (skipjack and bigeye, Table 4) showed the potential of this type of data to provide quantitative abundance estimations. This result was not surprising, as acoustics have long been a well-established methodology for estimating abundance of many pelagic fish species [21, 49]. Somewhat unexpected was the superior performance of the high frequencies (especially 200 kHz) and even the multifrequency synthetic average over the 38 kHz frequency to predict total abundance at a given DFAD (Table 4 and Fig 4). According to our results, use of the highest frequency (200 kHz) would be the best option in most of the cases due to its good performance on skipjack, the most abundant tuna species of the three (but at the cost of failing occasionally in cases of predominance of bigeye). Alternatively, the synthetic multifrequency average could constitute a robust compromise, being able to perform reasonably well in many proportion scenarios for the main tuna species.

The addition of the mean depth of the aggregation considerably improved the quantitative utilization of acoustic data. We interpret this as follows: we have the notion that tuna aggregations at DFADs tend to occupy the vertical range from ~20–25 m depth until a maximum depth, being this maximum depth proportional to the aggregated biomass (especially for large aggregations, where the maximum packing densities of tuna are expected to occur, and the higher the biomass, the more volume it occupies). That’s why we considered the mean depth as a valid proxy for the vertical extension of the aggregation. If the assumption was true, it would imply also the vertical extension of the aggregation to be proportional to the horizontal extension: the more biomass, the larger the volume. Even if the vertical extension of the aggregation is implicit in the NASC [36], the addition of the mean depth in the model adds information on its horizontal extent (supposed to be proportional to the vertical extent under an assumption of isotropy), thus improving the prediction of abundance. This result could be useful to improve abundance estimation in cases where the only source of information is the data from vertical echo-sounders (and there is a lack of information about the volume of the aggregation). The potential of the mean depth to improve abundance estimates could be tested in the algorithms used by the echo-sounder buoys used to track DFADs [50, 51].

Concerning the estimation of tuna species´ proportion found at DFADs, the significant relationship between mean frequency response and the proportions of each of the three tuna species (p < 0.001 for all, Table 4) also demonstrates potential for species discrimination. Although these relationships were expected for skipjack and bigeye, due to the clear patterns obtained for the DFADs with monospecific aggregations, the significant relationship between yellowfin and frequency response was not expected. This is due to both, the low proportion of yellowfin in the catches and because we have not been able to measure the frequency response of this species, yet. The case of yellowfin tuna is particularly interesting, as it shows a decreasing trend with ΔMVBS, i.e., similar to that of bigeye but with a flatter slope (Fig 5). Moreover, addition of yellowfin length to the model further increased agreement (Table 4). This result is in accordance with knowledge about late development of the swimbladder in yellowfin tuna, which has been reported to occur only after reaching ~45 cm length [37]. At the size ranges of yellowfin tuna present in this study, it would represent having ~90% of yellowfin tuna individuals with swimbladder. Hence, the dominant yellowfin bladdered fraction would cause decreasing frequency response observed at the DFADs we surveyed. Further, the contribution of the bladderless yellowfin fraction, and probably the smaller size of yellowfin bladder in general compared to bigeye [22], would cause the comparatively flat slope. More research would be necessary to properly test these hypotheses, combining TS measurements and backscattering simulations.

Despite the high significance of some of the modelling results obtained for prediction of abundance and species proportions, most of these relationships showed a considerable variability (Figs 4 and 5). Therefore, all these acoustic predictions will be accompanied by a degree of uncertainty. Some of this uncertainty might be reduced by using a more extensive set of acoustic parameters but probably some uncertainty will remain as part of the inherent variability of underwater acoustic measures. In this regard, the development of a library of acoustic samples versus catches for each region, would allow testing further proposed models and thus refining discrimination of tuna species by using the most appropriate acoustic parameters to explain percentage of the species found at DFADs for the different regions.

Finally, the acoustic parameters used in this work did not demonstrate good potential to predict mean tuna size. The only significant (p < 0.05) but weak relationship found between frequency response and tuna length, was probably because, generally, small tuna consists of skipjack and small yellowfin, i.e., without swimbladders, whereas large tuna consist of bigeye and large yellowfin, which have bladders, thus both groups providing a contrasting response.

It is important to note that tropical tunas are not the only fish species found at DFADs and some of the common by-catch species found at DFADs have large swimbladders and thus, strong acoustic backscattering, as is the case for pelagic triggerfish (*Canthidermis maculatus*) and rainbow runner (*Elagatis bipinnulata*), two species commonly found at DFADs. In order to discriminate them from tuna species, knowledge on the vertical distribution of the different species found at DFADs is necessary. Most non-tuna species that can cause confusion when acoustically determining presence and abundance of tunas at DFADs are found consistently shallower than target species [52]. Thus, in order to concentrate on tuna species presence and abundance on DFADs, targeting depths below that occupied by the non-target species may be an effective strategy. Although tuna species could also be found at shallower depths and some by-catch species can have a partially overlapping vertical distribution during some hours of the day, studies using tag sensors have shown that tuna species generally remain deeper than non-target species [53,54]. The few studies that have assessed the presence of tuna at DFADs using echo-sounders have not considered data above 25 m to avoid targeting non-tuna species [51, 55]. More effort is needed to study the natural behavior of tuna and other species at DFADs by region, using tags with pressure (depth) sensors would allow a better understanding of the vertical distribution of species at DFADs under different environmental conditions and thus allowing a more accurate vertical segregation of tuna and non-tuna species.

### Ongoing and upcoming research

In this work we have deliberately omitted an important acoustic-based parameter, the target strength or TS [27]. The TS is a measure of the acoustic response of a single fish, which is proportional to the length of the fish and is crucial to convert the echo-integrated acoustic measures into abundance through, e.g., *ρ* = *MVBS*/10^*TS*/10^, where *ρ* stands for volumetric density in number of fishes per m^3^. There are several reasons to omit TS from these analyses. First, it is more difficult to measure TS compared to obtaining echo-integration. A special transducer, the split beam, is needed to measure TS values and, so far, these transducers are not commonly available to the commercial purse seiner fleet, and until commonly available, it will be difficult to obtain TS-based measures onboard purse seiners and echo-sounder buoys used to track DFADs. In addition, even if a split beam echo-sounder is available, it is tricky to obtain an unbiased mean TS measure due to high risk of occurrence of unresolved multiple echoes. This has been recognized often in scientific literature [56, 57] and it requires rather sophisticated filters [58–60] to mitigate potential bias, especially in the large fish density conditions found in association with DFADs. In addition, these filters might affect differently, different species due to differences in size and behavior between them [61]. Therefore, in cases of mixtures of species, the computation of a mean TS of the aggregation might not be representative of the actual species composition. Therefore, we consider that, for now, it is more useful to investigate characterization of tuna based solely on echo-integration measures.

Of course, as we said, TS is crucial to estimate abundance and, as technology advances fast, it is likely that in a few years purse seiners and echo-sounder buoys will have split beam transducers available. In addition, in order to try to infer size of tuna based on acoustics, rather than echo-integration, we should extract TS-related information (which is proportional to fish length) from the acoustic data [27].

Therefore, there are several ongoing and upcoming lines of research concerning TS of the main tuna species at DFADs. Our most immediate goal is to study the frequency response and TS-length relationship of yellowfin and bigeye. For bigeye, we are in the process of publishing the results (Boyra et al., *pers*. *comm*.) while for yellowfin, due to the difficulty of finding mono-specific schools of this species at DFADs, we are planning to conduct these studies in an offshore cage in the IATTC Achotines Laboratory in Panama.

Once we know both echo-integration and TS-base frequency response for the 3 species, it should be possible to create a multifrequency acoustic mask to discriminate at least species into two groups: tunas with swimbladder (bigeye and large yellowfin) and tunas without swimbladder (skipjack and small yellowfin) and provide estimates (and measures of uncertainty) of their proportion at DFADs. In the medium term, our proposed procedure to characterize tuna from multifrequency narrowband acoustic data would comprise three steps. First, obtain proportions of swimbladder versus non-swimbladder fish based on MVBS-based frequency response. In a second step, a TS analysis should be done, after using multifrequency simultaneity requirements to filter multiple targets [60, 62]. This way, each TS value will be grouped according to its individual TS frequency response into bladdered or bladderless tuna and hence be used to roughly estimate size per group, applying approximate TS-length relationships per group. This would be a refinement over the work done by Moreno *et al*., 2008 concerning estimation and use of mixed TS at DFADs. Then, in the third step, an estimation of abundance could be done for each group using the surface backscattering density, vertical extension and TS using standard acoustic equations [27]. This type of procedure, although probably imprecise, has the benefit that it could be applied on remotely recorded acoustic data as those recorded by echo-sounder buoys used to track DFADs, and try to provide at least some rough, but objective size and species information in addition to abundance.

Last, as potential future lines of research, it is probably worth mentioning that the recent commercialization of split beam and broadband acoustic sensors [63], opens another possible lines of research on both species and size determination by taking advantage of the superior resolution and increased frequency response information provided by this type of acoustic sensors. In this regard, broadband acoustic data are being routinely collected in the cruises onboard tuna purse seiners and some (still preliminary) exploratory analyses are being conducted using machine learning techniques.

### Implementation of this knowledge on acoustic tools used by purse seiners

Results from this study suggest that vessels using low frequency echo-sounder buoys to track their DFADs (38 and/or 50kHz) may be more attracted to DFADs with higher proportion of tuna with swimbladders, i.e. yellowfin and bigeye tunas that are detected more strongly at lower frequencies.

It is not possible to quantify the implications of this, but an outcome might be adverse consequences for yellowfin and bigeye tuna stocks. This is since fishers plan visits to DFADs relying on the remote information on the strength of the acoustic signal and the acoustic signal coming from echo-sounder buoys using low frequencies would be stronger under the presence of yellowfin and bigeye. During worldwide skipper workshops held by ISSF, purse seine fishers have acknowledged the fact that they are not capable of discriminating tuna species using their available acoustic tools [19]. The fact that echo-sounders do not have information on the TS of the species present at DFADs as well as on their frequency response, results in many cases, that both the amount and species of fish found by fishers at DFADs is different from what was expected based on the acoustic information provided by buoys.

Authors of this study are working together with echo-sounder buoy manufacturers so that the new knowledge acquired in this research is incorporated in the acoustic equipment used by fishers in order to improve their discrimination skills when surveying DFADs. The ideal result would be to be able to remotely assess species composition using echo-sounder buoys attached to DFADs so that fishers could avoid lengthy transits and restrict their effort to areas with good concentrations of target species of desirable size. Additionally, it is common for fishers to set on natural floating debris or DFADs belonging to other vessels [64]. In those cases, the purse seiner making the set would not have remotely examined acoustic information of an echo-sounder buoy. The discrimination of species and/or sizes would be done using the acoustic equipment onboard.

However, it should be noted that improving knowledge of school abundance and composition through acoustics will likely result in increased fishing efficiency and might result in increased distance traveled by each vessel when they move from one DFAD to another.

### Acoustic discrimination to address undesired mortality of tuna and non-tuna species

Acoustic discrimination shall be particularly useful when different DFADs available to a vessel exhibit different proportions of species size and compositions. In other words, acoustic discrimination could be the key factor for a fisher to identify the most profitable and sustainable fishing by avoiding undesired species and by choosing among the diverse options available. If all DFADs were similar in terms of proportion and sizes of the 3 tropical tuna species, then the information obtained from the echo-sounder buoy would not have any value. However, it is well known that species composition and sizes vary among DFADs. These differences were evident not only during the research cruises described here but also in the literature on DFAD aggregations from the different oceans [9, 10].

Knowing relative biomass of each tuna species associated with a given DFAD alone will not be enough to allow fishers to make sustainable decisions. Incentives will also be necessary to achieve long-term conservation of tropical tunas. One of the potential incentives could be catch limits for bigeye and yellowfin. In some regions, as in the EPO there has been a common catch limit for bigeye and yellowfin tuna. Once a fisher, mainly fishing on DFADs, reaches the catch limit for those species they are obliged to stay in port due to the fact that DFAD aggregations are very unlikely to be mono-specific in skipjack and setting the net would cause the catch of bigeye or/and yellowfin. It is very costly to maintain and operate an industrial purse seiner, not only due to the high fuel cost but also in terms of fishers salaries, food, gear, and all the necessary logistics to keep up the boat in port and actively fishing at sea. The ability to select DFADs with a desirable species composition would allow vessels to plan and execute their fishing season in a profitable and ecologically sound manner. This strategy would be especially useful if vessel or fleet-specific quotas are enforced as opposed to fleet or fishery-wide regulations.

### Other uses of acoustic discrimination of tuna species

The importance of promoting fisheries that are managed for ecosystem health, not just a targeted species is clear but, as detailed by [65], there is a common concern of prohibitive data requirements for ecosystem-based management and the fear of expensive implementation. This statement is especially accurate for the case of tropical tunas that are found in offshore waters, as gathering *in situ* data on tunas and their pelagic ecosystem is expensive [31]. Likewise, tuna RFMOs in recent stock assessments for bigeye and yellowfin tuna, stated the lack of and need for fisheries independent estimates of tropical tuna abundance to reduce levels of uncertainty in current stock assessments. Acoustic instruments used by fishers could be the means to achieve data needs for scientists and selective fishing for fishers. Some fleets are already sharing with scientists, discrete data of echo-sounder buoys´ biomass estimates, however this data should be systematically recorded for the fleets operating with DFADs to obtain the needed spatial and temporal cover. European project “Strengthening Regional cooperation in the area of large pelagic fisheries data collection (RECOLAPE) is already working to develop alternative abundance indices in tropical tuna fisheries using echo-sounder buoy data.

There are still key unknowns related to the effect of DFADs on tuna behavior and mortality, having detailed data on the history of the DFAD (deployment, trajectory, soak-time) that could be related to the associated species´ biomass provided by echo-sounder buoys, would allow a better understanding of the processes driving tuna associative behavior with DFADs. Furthermore, a combined and simultaneous use of acoustic biomass by species and remote sensing oceanographic parameters, could serve to better understand the variation of the abundance, residence time and species composition of aggregations at DFADs related to the environment. Recent research efforts using echo-sounder buoy´s biomass estimations lack data on species composition, which hinders both a better estimate of biomass underneath DFADs and also species-specific studies [51, 66, 67]. Filling these knowledge gaps would allow designing sound science-based conservation measures, as a sustainable number of DFADs at sea and effective spatial and temporal closures.

## Conclusion

One of the main challenges currently faced by fishers working with DFADs is mitigating the adverse impacts of fishing on bigeye and yellowfin tuna, in areas where there is a need to reduce the fishing pressure on these species. There are few technological possibilities to avoid the catch of bigeye and yellowfin tuna at DFADs but that allow at the same time continue fishing on skipjack tuna. This manuscript provides first data on tropical tuna acoustic discrimination to date, that could be used for selective fishing with DFADs. Given the clearly distinct acoustic frequency responses found between skipjack and bigeye tuna, the potential and benefits of applying multi-frequency acoustics to discriminate species with swimbladder (yellowfin and bigeye) from species without swimbladder (skipjack) is confirmed. This positive result encourages further research to obtain the acoustic mask needed to determine the proportion of the 3 main tuna species found at DFADs. Next research steps include obtaining yellowfin tuna´s TS and frequency response. Improved discrimination results will become possible when a more extensive set of acoustic parameters are incorporated into the analysis to discriminate the 3 species. This information will be used as a set of characteristics to build an automatic classification model, based not only on volume integrated s_v_ but also on individualized fish TS frequency responses.

The present research also provides the means to uncover a new source of direct observation of tropical tuna species. Estimates are of 50,000–100,000 or more DFADs deployed every year globally [68]. Recently, some fleets have started sharing with scientist acoustic data provided by echo-sounder buoys attached to DFADs. Research efforts to use this information as new direct estimates of tuna abundance are ongoing, however data provided currently by fishers´ echo-sounder buoys comprises a single rough biomass estimate for all the species found at a given DFAD. The finding of the contrasting acoustic response of skipjack and bigeye tuna would allow obtaining, in the near future, more accurate biomass estimates by tuna species. This information is not only useful for selective fishing at DFADs. The collection of acoustic data provided by DFADs, through an appropriate collaborative scheme between fishers, scientists and buoy manufacturers, would be a significant step to fill current knowledge gaps on the abundance, distribution and behaviour of tuna at DFADs and thus support tropical tuna conservation.
